# Alleviation of Autophagic Deficits and Neuroinflammation by Histamine H3 Receptor Antagonist E159 Ameliorates Autism-Related Behaviors in *BTBR* Mice

**DOI:** 10.3390/ph17101293

**Published:** 2024-09-28

**Authors:** Shilu Deepa Thomas, Petrilla Jayaprakash, Nurfirzana Z. H. J. Marwan, Ezzatul A. B. A. Aziz, Kamil Kuder, Dorota Łażewska, Katarzyna Kieć-Kononowicz, Bassem Sadek

**Affiliations:** 1 Department of Pharmacology & Therapeutics, College of Medicine and Health Sciences, United Arab Emirates University, Al Ain P.O. Box 17666, United Arab Emirates; 700039712@uaeu.ac.ae (S.D.T.); petrilla.jp@uaeu.ac.ae (P.J.); firzana.hjm@gmail.com (N.Z.H.J.M.); ezzatulaqilahaziz@gmail.com (E.A.B.A.A.); 2 Zayed Center for Health Sciences, United Arab Emirates University, Al Ain P.O. Box 15551, United Arab Emirates; 3 Department of Technology and Biotechnology of Drugs, Faculty of Pharmacy, Jagiellonian University Medical College, Medyczna Str. 9, 30-688 Kraków, Poland; kamil.kuder@uj.edu.pl (K.K.); dorota.lazewska@uj.edu.pl (D.Ł.); mfkonono@cyf-kr.edu.pl (K.K.-K.)

**Keywords:** autism spectrum disorder, H3 receptor antagonists, repetitive behavior, social features, neuroinflammation, autophagy, mTOR, *BTBR* mice

## Abstract

Background/Objectives: Autism spectrum disorder (ASD) is a neurodevelopmental condition marked by social interaction difficulties, repetitive behaviors, and immune dysregulation with elevated pro-inflammatory markers. Autophagic deficiency also contributes to social behavior deficits in ASD. Histamine H3 receptor (H3R) antagonism is a potential treatment strategy for brain disorders with features overlapping ASD, such as schizophrenia and Alzheimer’s disease. Methods: This study investigated the effects of sub-chronic systemic treatment with the H3R antagonist E159 on social deficits, repetitive behaviors, neuroinflammation, and autophagic disruption in male *BTBR* mice. Results: E159 (2.5, 5, and 10 mg/kg, i.p.) improved stereotypic repetitive behavior by reducing self-grooming time and enhancing spontaneous alternation in addition to attenuating social deficits. It also decreased pro-inflammatory cytokines in the cerebellum and hippocampus of treated *BTBR* mice. In *BTBR* mice, reduced expression of autophagy-related proteins LC3A/B and Beclin 1 was observed, which was elevated following treatment with E159, attenuating the disruption in autophagy. The co-administration with the H3R agonist MHA (10 mg/kg, i.p.) reversed these effects, highlighting the role of histaminergic neurotransmission in observed behavioral improvements. Conclusions: These preliminary findings suggest the therapeutic potential of H3R antagonists in targeting neuroinflammation and autophagic disruption to improve ASD-like behaviors.

## 1. Introduction

Autism Spectrum Disorder (ASD) is characterized by challenges in social communication, and stereotypic and repetitive behaviors, affecting learning and development [1]. ASD frequently co-occurs with other conditions, such as depression, epilepsy, tics, attention-deficit hyperactivity disorder, sleep disorders, and gastrointestinal issues [2,3]. Despite its prevalence and impact, effective treatments for core symptoms are lacking, highlighting the need for more precise and efficient therapies [4].

Various factors such as oxidative stress, neuroimmune dysfunction, imbalances in excitatory and inhibitory neurotransmitters, and deficits in neurotrophic factors are implicated in the pathogenesis of ASD [5,6,7,8]. Inflammation and neuroimmune dysregulation are critical in ASD [6,9,10]. Exposure to inflammation during pregnancy leads to disruption of normal fetal neuronal development, resulting in irregular neuronal activity, social behavior changes, and poor cognitive performance in the offspring [11,12]. Neuroinflammation, closely linked to cognitive impairment, involves microglia, the brain’s immune cells, releasing proinflammatory cytokines that are harmful to neurons and perpetuate inflammation [13,14].

Autophagy is a cell process crucial for maintaining internal homeostasis by eliminating damaged proteins, which affects axonal maintenance, synaptic pruning, and neurogenesis. Furthermore, evidence suggests that autophagy can also modulate microglial activation [15]. Autophagy reduces inflammation and prevents cell death [16,17]. It is controlled by the mTOR complex, which is crucial for energy metabolism, protein synthesis, as well as cell growth [18]. According to a study by Lieberman et al. (2020), autophagy is downregulated during postnatal development following the upregulation of mTOR in the mice. They also found that during late postnatal development, impairments in autophagy are associated with deficiencies in synaptic transmission and social behavior [19]. Multiple lines of evidence show disruption in autophagic pathways in ASD animal models suggesting a potential role in the pathogenesis of ASD [20,21].

The histamine H3 receptor (H3R) is a constitutively active receptor primarily located in the brain, functioning as a presynaptic autoreceptor that inhibits histamine synthesis and release [22]. H3Rs also function as heteroreceptors, modulating the release of various neurotransmitters such as acetylcholine (Ach), gamma-aminobutyric acid (GABA), serotonin (5-HT), and dopamine (DA). Histamine has also been shown to influence behaviors in disorders that overlap with ASD, such as Alzheimer’s disease, schizophrenia, and Tourette’s. Multiple studies have connected histaminergic signaling through H3Rs to autism-like repetitive behaviors. This connection is supported by post-mortem brain analyses, genetic association studies, and animal models [23,24,25].

Preclinical and clinical studies suggest that antagonizing H3Rs can alleviate behavioral and cognitive symptoms in Alzheimer’s disease and schizophrenia, both of which share similar behavioral features with ASD, including cognitive impairment [26]. H3R antagonism improved performance in context discrimination tasks in aged mice [27]. It has also been shown to improve cognitive deficits induced by NMDA receptor antagonists like MK-801 and ketamine [28]. In an animal model of schizophrenia, an H3R antagonist improved behavioral deficits as well as spatial working memory [29], which are also observed in patients with ASD [30]. Furthermore, antagonizing H3R mitigated impairment in social behavior in animals exposed to phencyclidine, an outcome that may be relevant for ASD [31]. Notably, H3R antagonist ciproxifan was found to reduce stereotypies and sociability deficits in a rodent model of autism [23]. The non-imidazole-based H3R antagonist DL77 has been reported to exhibit promising effects in improving autism-like features induced by valproic acid in mice [32]. Additionally, dual-action H3R antagonist and acetylcholinesterase (AChE) inhibitor E100 reduced oxidative stress and proinflammatory cytokine levels, while also alleviating social impairments and repetitive stereotypical behaviors in C57BL/6 mice exposed to valproic acid [33]. Several H3R antagonists have shown promise in bringing behavioral improvements in ASD by targeting neuroinflammation [34]. The H3R antagonist ST713 reduced NF-κB activity and cytokine levels (TNF-α, IL-1, and IL-6) in *BTBR* mice, a model of autism [35]. These studies highlight the therapeutic potential of H3R antagonists in managing both cognitive and social impairments observed in ASD. Notably, H3R antagonism has been linked to enhanced autophagy via the PI3K/AKT/mTOR pathway, offering protection against ischemic injury [36].

**Figure 1:**
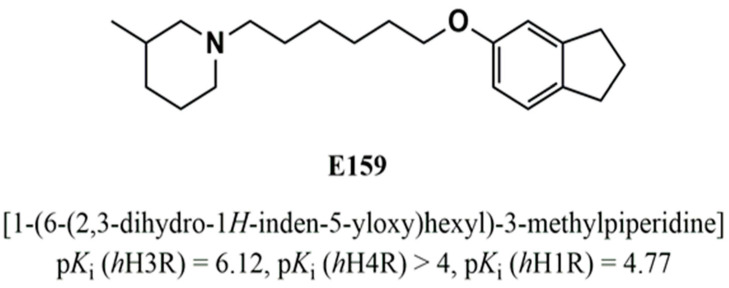
Chemical structure and pharmacological in vitro binding affinity profile of E159 on selected human histamine receptor subtypes.

The *BTBR T+ Itpr3tf/J (BTBR)* inbred mouse strain, presents ASD-related behaviors with reduced sociability and increased repetitive behaviors, like marble burying and self-grooming [37,38]. In the current study, the test compound E159 and its docking to human H3R were evaluated using in silico methods. Subsequently, we evaluated the impact of systemic sub-chronic treatment with E159, a potent and selective H3R antagonist with high in vitro specificity for H3Rs (Figure 1), on ASD-related behavioral dysfunctions in *BTBR* mice [39]. The H3R antagonist E159 was selected based on its previous significant procognitive effects in a model of memory deficit induced by dizocilpine in rodents [40]. Additionally, the effects of the sub-chronic treatment of E159 on neuroinflammation and autophagy in the cerebellum of treated mice were evaluated. Also, the effects of E159 on repetitive and social behavioral deficits were assessed in *C57BL/6J (B6)* which served as the control strain, displaying normal sociability and a low level of repetitive behaviors [41,42]. The H3R plays a key role in regulating the release of neurotransmitters, particularly histamine. (*R*)-α-methylhistamine (MHA) is a CNS-penetrant and selective H3R agonist [32]. To further explore the involvement of histaminergic signaling in the behavioral effects observed for H3R antagonist E159, we investigated whether the agonist MHA could counteract the positive effect of H3R antagonist E159. The abrogation study assessed whether sub-chronic systemic co-administration of the CNS-penetrant H3R agonist MHA could reverse the behavioral and biochemical improvements elicited by E159. This approach aimed to clarify the role of brain histaminergic neurotransmission in mediating the beneficial effects of E159.

## 2. Results

### 2.1. Docking Studies of E159

For in silico studies, receptor-ligand complexes for three histamine receptors, H1, H3, and H4, were chosen, which are represented by PBD structures 3RZE [43], 7F61 [44], and 7YFC [45], respectively. Docking to the H1R structure resulted in a putative calculated pose (dG Bind = −51.42 kcal/mol), where the protonated nitrogen formed a salt bridge with D107^3.32^ and cation-π with Y108^333^. The proton (at nitrogen) was directed toward TM6, and the whole ligand bent in a way that the distal aromatic group was parallel to TM5 with no additional interactions. On the other hand, docking to H4 structures resulted in low-scoring poses with dG Bind = 20.88 kcal/mol, which may explain the low affinity for both targets.

**Figure 2:**
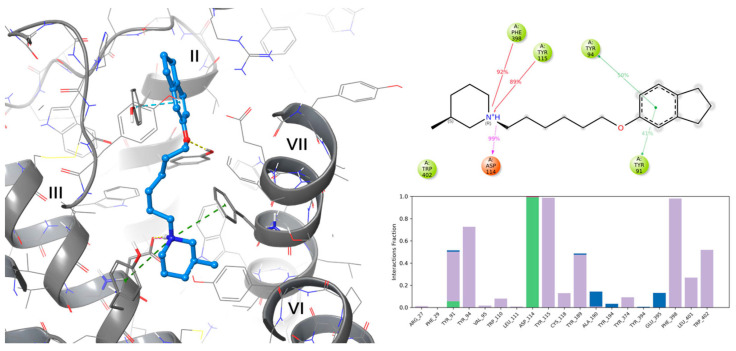
**Left** panel: Predicted binding mode of E159 (**left**) within histamine H3R receptor binding site. Hydrogen bonds are shown with yellow dashed lines, salt bridges with magenta lines, cation-π interactions with green lines, and π−π interactions with blue lines. Roman numerals indicate the respective TMs; **Right** panel: Summary of ligand-protein contacts from MD simulation (**top**; hydrogen bond is shown with a purple dashed line, π−π interactions as green and cation-π as red lines), and contacts histogram (**bottom**; green for hydrogen bonds, violet for hydrophobic contacts, blue for water bridges; X-axis represents interaction fraction (1.0 = 100% simulation time), Y-axis represents particular interacting amino acids.

In the case of the H*3*R complex, the compound exhibited a relatively high binding free energy (dG Bind) of −91.43 kcal/mol. It occupied the H*3*R binding pocket in a manner similar to the re-solved complex ligand PF03654746, maintaining key interactions typical of histamine H*3*R antagonists/inverse agonists. These interactions included the formation of a salt bridge and a hydrogen bond between the protonated amine nitrogen and D114^3.32^. Additional ligand’s west-end stabilization through cation-π interactions with caging, aromatic sidechains of Y115^3.33^, and F398^7.39^ was also found, while piperidine 3-methyl substituent occupied the cavity formed by Y374^6.51^ and W402^7.43^ at the sides and F398^7.36^ at the top. The east-end aromatic substituent of E159 was positioned within the space enclosed by the aromatic features of Y189 (ECL2) above and Y91^2.61^ & Y94^2.64^ on the sides, further stabilizing the structure through either hydrogen bonding with the ether oxygen or π-π stacking interactions, respectively (Figure 2).

**Figure 3:**
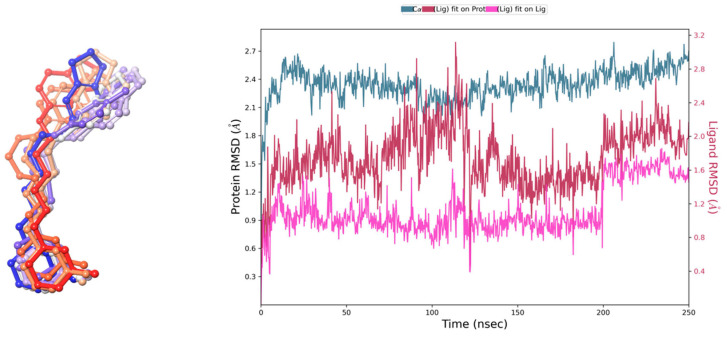
(**Left** panel): Orientation of E159 during the 250 ns MD simulation. Different colors represent distinct frames: 0 ns is shown in blue, transitioning through the violet spectrum (dark to light: 25–100 ns) to grey (125 ns) and vice versa through the orange spectrum (light to dark: 150–225 ns) to red (250 ns). (**Right** panel): Time evolution of RMSD for ligand (magenta) and protein (grey blue and dark red) for specific frames relative to the reference frame at 0 ns.

The stability of the calculated pose was assessed through 250 ns molecular dynamics (MD) simulations. Ten snapshots were selected from the simulation starting pose and after each 25 ns (Figure 3). Analysis of the ligand position during the simulation revealed that E159 remained stable throughout most of the recorded trajectory time, maintaining crucial interactions (Figure 2), and consistently engaging with a set of crucial amino acids, although the final conformation was slightly varied from its starting orientation Most of the interactions occur within the 2nd, 3rd and 7th helices. However, the highly flexible nature of the alkyl spacer and the distal aromatic group was also observed which resulted in fluctuations of RMSD value. Around 200 ns the aromatic feature moved closer to Y94^2.64^, losing the support from the other tyrosine moiety (Y91^2.61^) in favor of Y94, followed by a slight drop in complex binding free energy to the level of −80.66 kcal/mol. Yet, overall, the ligand displayed stable key interactions throughout the whole time of the recorded simulation, suggesting that the ligand might stabilize the inactive state of the receptor. Last but not least, the postulated H_3_R inactive state 3–7 lock between D114^3.32^ and W402^7.43^ [46] was observed through the whole recorded trajectory, which may give additional confirmation of the presumed stabilization of the inactive state of the receptor by the tested E159 ligand.

### 2.2. Effects of E159 on Stereotypical Repetitive Self-Grooming Behaviors in Mice

**Figure 4:**
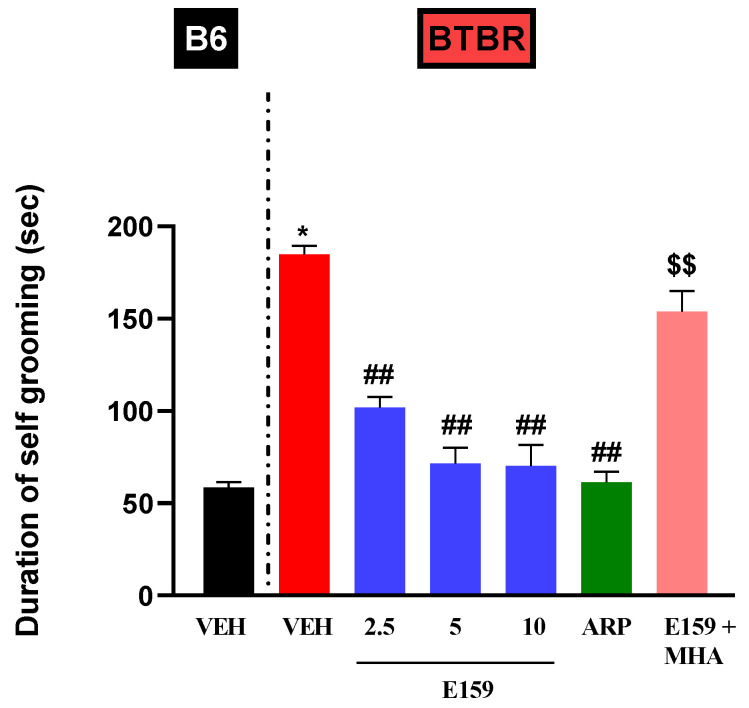
E159 mitigated compulsive grooming in *BTBR* mice. Both *B6* and *BTBR* mice, were administered an intraperitoneal injection of a vehicle, E159 or ARP before the assessment of self-grooming. *BTBR* mice exhibited a significantly higher grooming duration in comparison to *B6* mice. E159 as well as ARP, significantly decreased self-grooming in the autistic model. Additionally, the impact of co-injection of (R)-α-methylhistamine (MHA) on E159 (2.5 mg/kg)-induced reduction in grooming duration in *BTBR* mice was evaluated. * *p* < 0.001 relative to *B6* mice treated with vehicle, ^##^ *p* < 0.001 relative to *BTBR* mice treated with vehicle, ^$$^ *p* < 0.01 relative to E159 (2.5 mg)-treated autistic mice, (n = 6). (mean ± SEM, n = 6/group).

The impact of various treatments on mouse grooming duration is depicted in Figure 4. Statistical analyses showed substantial effects for strain, treatment, and their interaction (*p* < 0.001). The systemic pretreatments with E159 (2.5, 5, and 10 mg/kg) and ARP (1 mg/kg) did not alter the self-grooming behavior in *B6* mice (Table 1). Statistical analyses demonstrated that VEH-treated *BTBR* mice (184.7 ± 4.752 s) spent considerably more time grooming than *B6* mice (58.5 ± 2.89 s) (*p* < 0.001). The compound E159 at all the tested doses significantly reduced grooming duration (all *p*’s < 0.001) in autistic mice. ARP (1 mg/kg, i.p.) also displayed a considerable reduction in self-grooming duration in *BTBR* mice (*p* < 0.001). As shown in Figure 4, the reduction in self-grooming time induced by E159 (2.5 mg) was prevented upon co-treatment with MHA (10 mg/kg) (*p* < 0.01), relative to *BTBR* mice administered 2.5 mg of E159 alone (Figure 4).

### 2.3. Effects of E159 on Spontaneous Alternation in BTBR Mice

**Figure 5:**
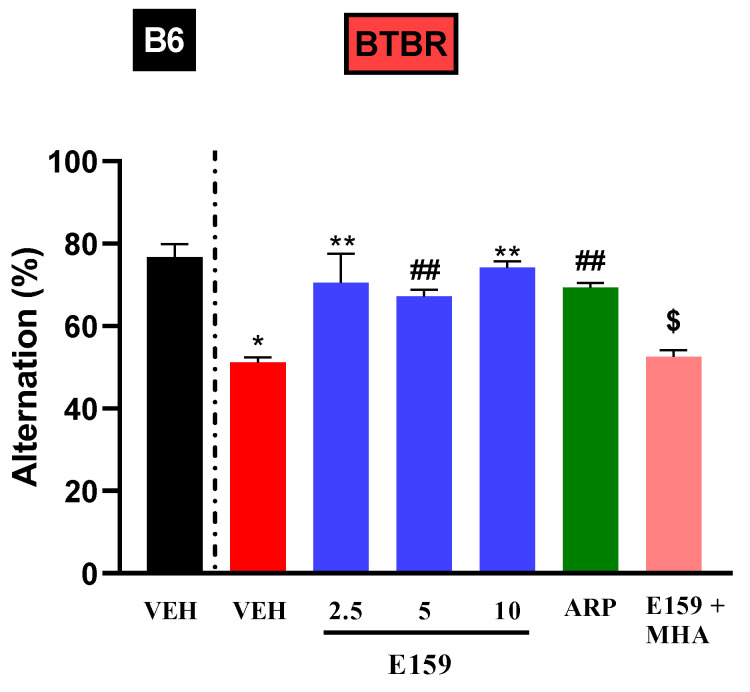
E159 treatment enhanced the alternation behavior in autistic mice. A significantly lower alternation behavior was seen in *BTBR* mice compared to *B6* mice. However, E159 or ARP considerably improved the alternation in *BTBR* mice. The impact of MHA (10 mg/kg) co-administration on the enhancement of alternation behavior induced by E159 (2.5 mg) in autistic mice was evaluated. * *p* < 0.001 relative to *B6* mice treated with vehicle. ** *p* < 0.01, ^##^ *p* < 0.05 relative to *BTBR* mice treated with vehicle. ^$^ *p* < 0.01 relative to E159 (2.5 mg/kg, i.p.)-treated *BTBR* mice. (mean ± SEM, n = 6/group).

Statistical analyses revealed substantial main effects for strain (F_(1,60)_ = 25.63, *p* < 0.001), treatment (F_(5,60)_ = 5.30, *p* < 0.01), and their interaction (*p* < 0.01). The vehicle-treated *BTBR* animals showed considerably low spontaneous alternation relative to control *B6* mice (*p* < 0.001) (Figure 5). Treatment with E159 (2.5, 5, and 10 mg/kg, i.p.) and ARP (1 mg/kg, i.p.) significantly increased the spontaneous alternation percentage in *BTBR* mice, with F-values of (F_(1,10)_ = 7.12, *p* < 0.01), (F_(1,10)_ = 63.8, *p* < 0.05), (F_(1,10)_ = 130.96, *p* < 0.01) and (F_(1,10)_ = 106.4, *p* < 0.05) respectively. Furthermore, no significant differences were found between the tested doses of E159 (all *p* > 0.05). In *B6* mice, E159 (2.5, 5, and 10 mg/kg, i.p.) and ARP (1 mg/kg, i.p.) did not affect the spontaneous alternation percentage (Table 1). Moreover, the enhancement in alternation seen with E159 (2.5 mg) in autistic mice was completely negated by simultaneous administration with MHA (*p* < 0.01) in comparison with *BTBR* animals treated with E159 (2.5 mg) alone (Figure 5).

### 2.4. Effects of E159 on Sociability and Social Novelty Preference of BTBR Mice in the Three-Chambered Task

**Figure 6:**
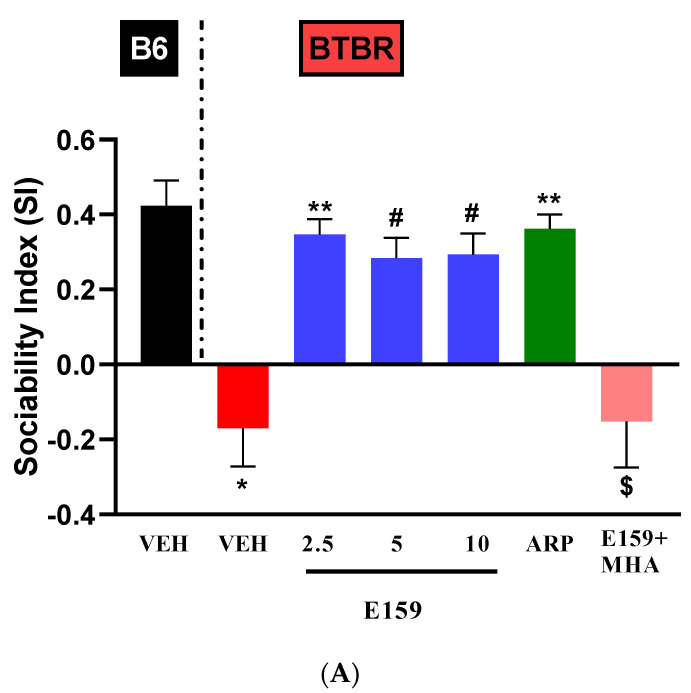

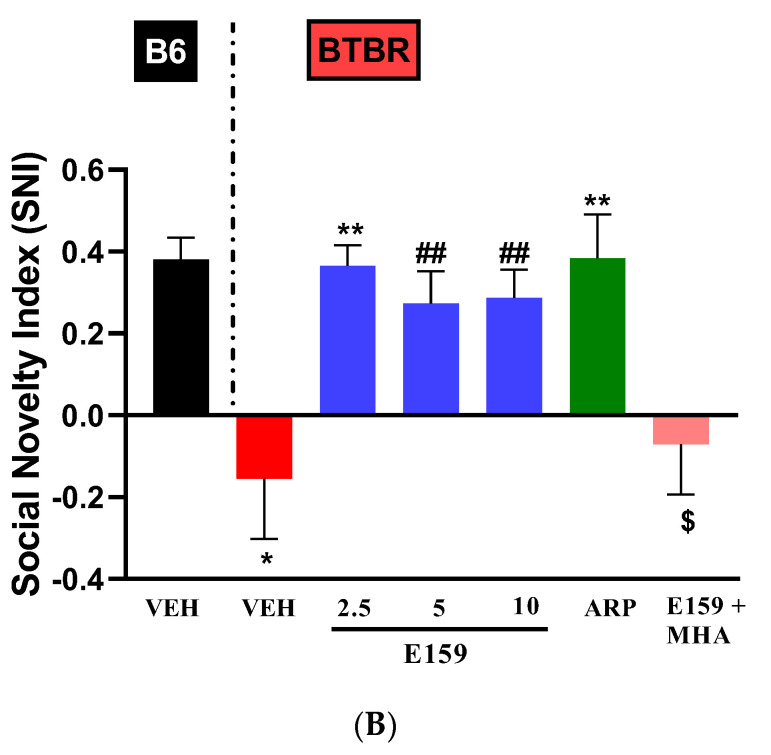
E159 improved impaired sociability in the autistic mice. Mice explored all three chambers for two consecutive 10 min sessions. Results measured included (**A**) the Sociability Index (SI) and (**B**) the Social Novelty Index (SNI). *BTBR* mice were administered E159 or ARP. The impact of co-administering MHA (10 mg/kg) on the enhancement of SI and SNI induced by 2.5 mg of E159 in the autistic model was evaluated. (**A**) SI: * *p* < 0.001 relative to *B6* mice treated with vehicle. ** *p* < 0.001, ^#^ *p* < 0.01 relative to *BTBR* mice treated with vehicle, ^$^ *p* < 0.05 relative to E159 (2.5 mg)-treated *BTBR* mice. (**B**) SNI: * *p* < 0.01 relative to *B6* mice treated with vehicle, ** *p* < 0.01, ^##^ *p* < 0.05 relative to *BTBR* mice treated with vehicle, ^$^ *p* < 0.01 relative to E159 (2.5 mg)-treated *BTBR* mice, (n = 6). (mean ± SEM, n = 6/group).

The effects of sub-chronic systemic administration of vehicle, E159 (2.5, 5, and 10 mg/kg, i.p.) and ARP (1 mg/kg, i.p.) on sociability deficits (SI) in *BTBR* mice in the three-chamber paradigm are depicted in Figure 6A. The data analyses exhibited a substantial impact for strain, treatment, and their interaction (*p*’s < 0.01). The *BTBR* mice exhibited a very low percentage of Sociability Index (SI) versus *B6* mice (F_(1,10)_ = 23.03, *p* < 0.001). E159 (2.5, 5, and 10 mg/kg) markedly improved the sociability of our autistic model (*p* < 0.01). ARP also considerably enhanced the sociability of *BTBR* mice (*p* < 0.01). The results also indicated that improvement in SI with E159 (2.5 mg/kg) was statistically comparable to that with ARP (*p* > 0.99). Additionally, the sociability-enhancing effects of E159 (2.5 mg) were nullified by co-treatment with MHA, H3R agonist (F_(1,10)_ = 5.72, *p* < 0.05). Furthermore, systemic pretreatment with E159 (2.5, 5, and 10 mg/kg) or ARP (1 mg/kg) did not alter SI in *B6* control mice in the three-chamber task (Table 1). Similarly, the effects of sub-chronic systemic administration of E159 (2.5, 5, and 10 mg/kg, i.p.) and ARP (1 mg/kg, i.p.) on social novelty preference were evaluated in *BTBR* mouse model (Figure 6B). In a two-way ANOVA, both strain and treatment had significant effects, along with a significant strain × treatment interaction (*p* < 0.05). A considerably lower percentage of Social Novelty Index (SNI) in *BTBR* animals relative to *B6* mice (*p* < 0.01) was observed. E159 (at all tested doses) appreciably increased SNI versus vehicle administered autistic mice (*p* < 0.05) (Figure 6B). Similar to SI results, ARP also improved SNI in *BTBR* animals relative to *BTBR* animals that received vehicle (*p* < 0.01). Furthermore, effects of E159 (2.5 mg) on SNI were entirely abolished by co-treatment with MHA [(F_(1,10)_ = 10.57, *p* < 0.01)]. Notably, systemic pretreatment with E159 (2.5, 5, and 10 mg/kg) or ARP (1 mg/kg) did not alter SNI in *B6* mice (Table 1).

### 2.5. Impact of E159 on Anxiety and Locomotor Activity

**Figure 7:**
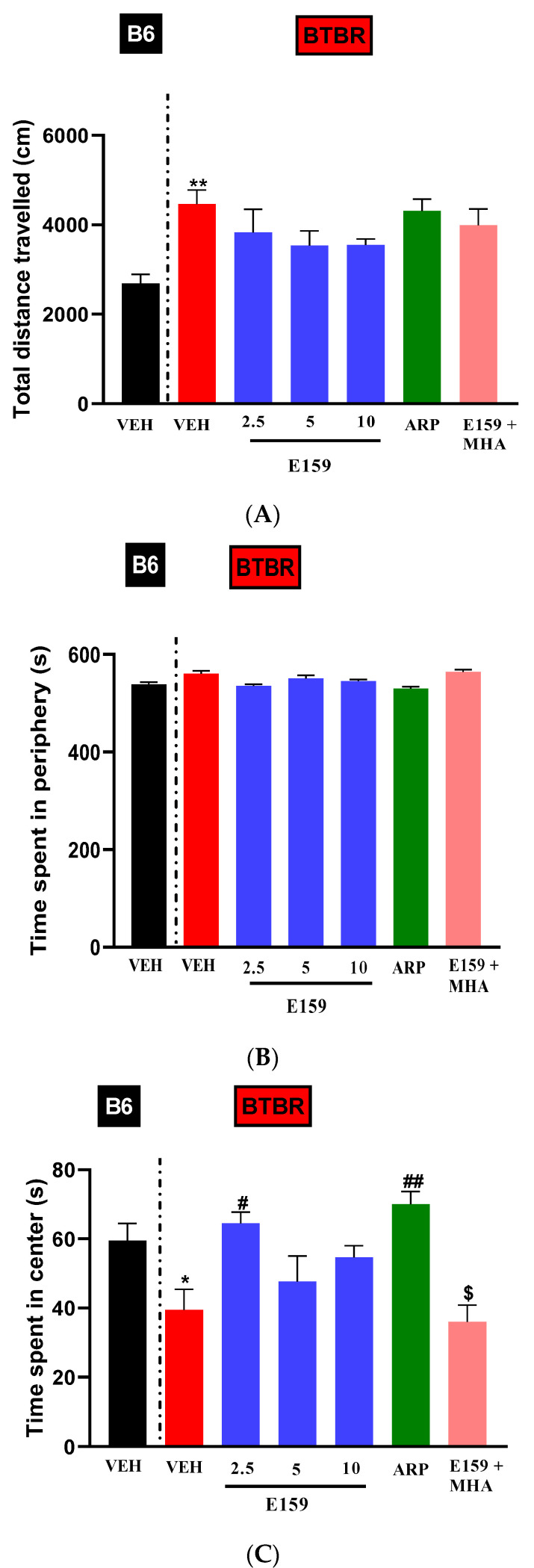
Treatment with E159 had no discernible impact on locomotor ability in autistic mice. (**A**) *BTBR* mice showed significantly greater distances travelled relative to *B6* mice. (**B**) Pretreatment with E159 or ARP did not significantly affect the duration spent in periphery in autistic mice. (**C**) Additionally, *BTBR* mice spent less duration in the center relative to *B6* mice. Data are shown as mean ± SEM. * *p* < 0.05, ** *p* < 0.01 relative to *B6* mice treated with vehicle. ^#^ *p* < 0.05, ^##^ *p* < 0.01 relative to *BTBR* mice treated with vehicle. ^$^ *p* < 0.01 versus *BTBR* mice treated with E159 (2.5 mg) (n = 6).

The open field test (OFT) evaluated locomotor ability and anxiety in both assessed strains (Figure 7). Two-way ANOVA results for the travelled distance exhibited substantial effect only for the strain (F_(1,60)_ = 77.40, *p* < 0.001) (Figure 7A). The *BTBR* mice that received vehicle treatment travelled significantly more than the *B6* mice that received the same treatment. (F_(1,10)_ = 22.27, *p* < 0.01). Pretreatment with E159 (2.5, 5, and 10 mg/kg, i.p.) or ARP (1 mg/kg, i.p.) did not significantly alter the total distance travelled in either *B6* or *BTBR* mice (Figure 7A, Table 1).

Figure 7B depicts the time spent in the periphery during the OFT following the systemic injections of the vehicle, E159 or ARP. There were no significant effects for strain and treatment (all *p*’s > 0.05), but significant effect was observed for strain × treatment interaction (F_(5,60)_ = 2.581, *p* < 0.05). Pretreatment with E159 (2.5, 5, and 10 mg/kg, i.p.) or ARP (1 mg/kg, i.p.) did not significantly alter the time spent in periphery in either *B6* or *BTBR* mice (Figure 7B, Table 1).

Figure 7C shows time spent in the central arena during the OFT after treatment with vehicle, E159, or ARP in *B6* and *BTBR* animals. The two-way ANOVA revealed considerable effect for strain, treatment, and strain × treatment interaction (*p* < 0.05). Vehicle administered *BTBR* animals spent significantly reduced duration in the central zone versus *B6* animals (F_(1,10)_ = 6.59, *p* < 0.05). E159 (2.5 mg/kg) and ARP considerably improved the duration in the central arena by *BTBR* mice (*p* < 0.05), whereas E159 (5 and 10 mg/kg) did not produce significant effects (all *p*’s > 0.05). In *B6* mice, time in the central arena was not altered by any of the treatments (Table 1). Notably, the E159 (2.5 mg)-induced increase in center time was decreased by simultaneous administration of MHA (F_(1,10)_ = 23.69; *p* < 0.01).

### 2.6. Effects of E159 on the Level of Proinflammatory Cytokines in Cerebellum and Hippocampus of BTBR Mice

Statistical analyses showed that there was a considerable elevation in all three proinflammatory cytokines in autistic mice relative to *B6* mice (*p* < 0.001). E159 at a dose of 2.5 mg/kg alleviated rise in proinflammatory cytokines in *BTBR* animals. The cytokines were measured in hippocampal and cerebellar tissues (Table 2). The cerebellar levels of cytokines showed considerable decline after systemic pretreatment with E159 (all *p* < 0.05). Similar decline in inflammatory response was also observed in hippocampus (*p* < 0.01) with reduced TNF-α, IL-6, and IL-1β. In addition, ARP notably reduced proinflammatory cytokines in *BTBR* animal strain (all *p* < 0.05). Additionally, the beneficial effects of E159 on cerebellar and hippocampal cytokines were prevented upon co-administration with (R)-α-methylhistamine (MHA) (*p* < 0.05) (Table 2).

### 2.7. Effect of E159 on Autophagy

**Figure 8:**
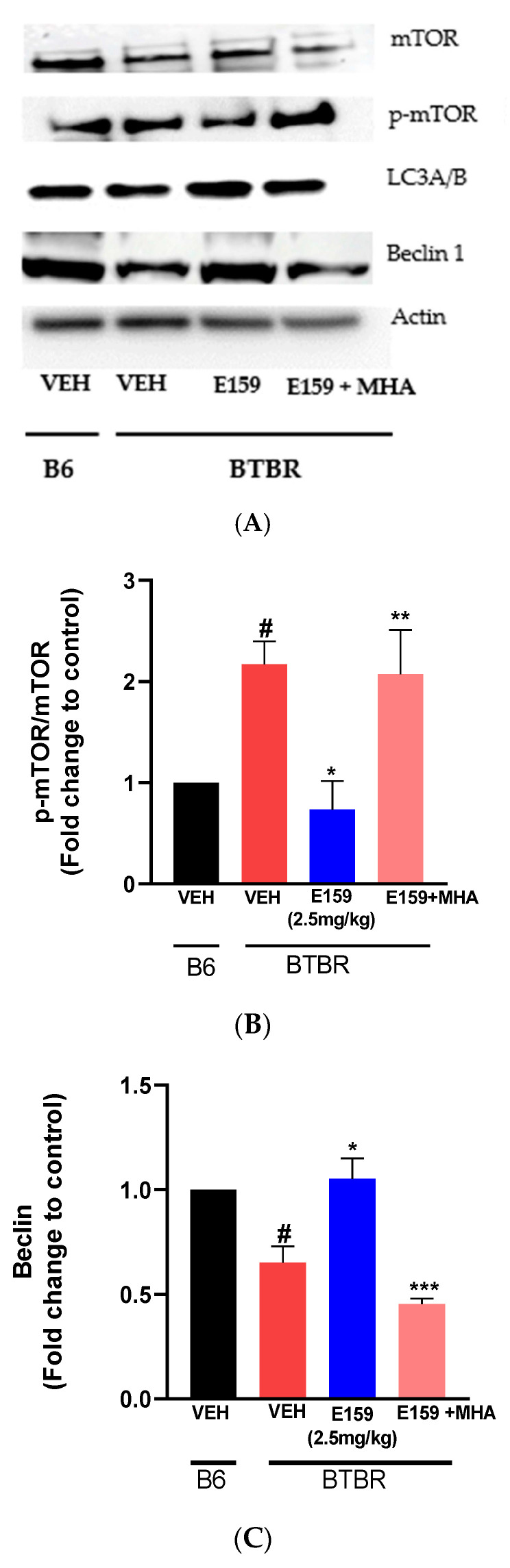

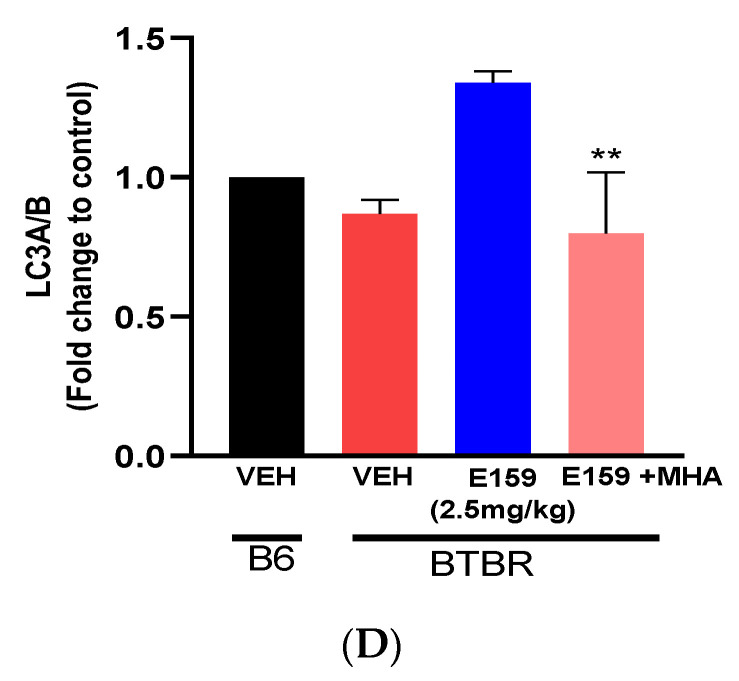
The proteins p-mTOR, mTOR, LC3A/B and Beclin-1 were determined by Western blotting (**A**). *BTBR* mice showed decreased expression level of Beclin-1 and LC3A/B in the cerebellum with increase in the levels of p-mTOR/ mTOR which further suggests autophagic deficiency. E159 ameliorates autophagic deficits with reduced levels of p-mTOR/ mTOR (**B**), and increased Beclin-1 (**C**) and LC3A/B (**D**). ^#^ *p* < 0.05 versus *B6* mice treated with vehicle. * *p* < 0.05 versus *BTBR* mice treated with vehicle. ** *p* < 0.05,*** *p* < 0.01 versus E159 (2.5 mg)-treated *BTBR* mice (n = 3).

The impact of sub-chronic injections of the E159 (2.5 mg/kg) on the autophagic proteins in autistic *BTBR* strain is illustrated in Figure 8. The proteins mTOR, p-mTOR, LC3, and Beclin 1 in the cerebellum of *BTBR* mice were detected by Western blotting (Figure 8A). The mean protein expression in *B6* mice was set as a fold change of 1 on graph. Densitometric analysis of cerebellar tissues from vehicle *BTBR* mice showed significantly elevated level of p-mTOR/ mTOR (Figure 8B) relative to *B6* mice treated with vehicle (*p* < 0.05). Nevertheless, administration of E159 (2.5 mg/kg) significantly decreased p-mTOR in the cerebellum of E159 treated *BTBR* mice, which further indicates the inhibition of mTOR activity and activation of autophagy. This reduction in protein expression was reversed upon co-injection with H3R agonist MHA (10 mg/kg). Furthermore, a significant decline in levels of Beclin 1 and LC3 (A/B) in cerebellum of autistic strain relative to the *B6* strain was observed, indicating impairment of autophagy (*p* < 0.05) (Figure 8C,D). The systemic sub-chronic treatment with E159 (2.5 mg/kg) provided elevation in the LC3 (A/B) and Beclin 1 levels in *BTBR* mice. Furthermore, the increase in the levels of LC3 (A/B) and Beclin 1 brought about by E159 (2.5 mg/kg) treatment, was reversed with the administration of MHA, an H3R agonist.

## 3. Discussion

ASD is characterized by deficits in sociability and repetitive behaviors, with histamine acting through H3 receptors (H3Rs) influencing functions like circadian rhythms and sensory sensitivity, which overlap with ASD symptoms [26,47]. Studies have linked histaminergic signaling via H3Rs to autism-like repetitive behaviors, supported by post-mortem brain analyses, genetic studies, and animal models. Disruptions in histaminergic signaling are also implicated in Tourette syndrome, a disorder associated with stereotypies similar to those in ASD [48,49]. H3R antagonists have shown promise in reducing ASD-like behaviors in several animal models, suggesting their potential therapeutic benefit [23,33].

*BTBR* mice serve as an idiopathic model of ASD due to their autism-like symptoms, such as repetitive behaviors and reduced sociability [41]. Studies suggest immunological changes in ASD models are linked to abnormal central nervous system development and ASD-like behaviors [50]. Altered immune profiles affect repetitive behaviors and social interactions in *BTBR* mice [51,52]. *BTBR* mice were selected for this study because they exhibit behavioral and inflammatory profiles similar to human ASD, including social deficits and abnormal self-grooming, unlike the highly sociable and normally grooming *C57BL/6J (B6)* mice [35,53]. This study evaluated the impact of the highly selective and potent H3R antagonist E159 on autism-like behaviors in *BTBR* mice and examined the effects of sub-chronic treatment with E159 on pro-inflammatory cytokines in cerebellum and hippocampus of treated mice. Also, the study aimed to investigate the effect of E159 on the expression of autophagic proteins LC3A/B and Beclin 1 in the cerebellum of treated mice.

In line with several previous studies, *BTBR* mice showed significant grooming compared to *B6* mice [54]. Rapanelli et al. (2017) have reported that repetitive behaviors in mice arise as a result of brain histamine deficiency [49,55]. E159 (2.5, 5, 10 mg/kg, i.p.) treatment dose dependently ameliorated this repetitive stereotypic behavior in *BTBR* mice effectively, which was comparable to the effect of ARP (1 mg/kg, i.p). Spontaneous alternation or the Y-maze test relies on animals’ normal tendency to explore new environments. In addition to the assessment of repetitive behaviors, it also tests the spatial working memory in mice [56]. Animals with impaired memory repeatedly enter the previously explored arm, showing fewer spontaneous alternations. Similar to previous studies, *BTBR* mice demonstrated reduced alternation compared to *B6* mice, reflecting attentional and cognition deficits [57]. Our results showed that E159 significantly improved spontaneous alternation in *BTBR* mice, which further confirms that E159 treatment can restore impaired short-term memory. This aligns with a previous study where the Y-maze test shows an augmented spontaneous-alternation rate following treatment with H3R antagonist thioperamide in a model of LPS-induced neuroinflammation [58]. The reference drug ARP also significantly reduced self-grooming as well as enhanced the percentage of alternation in *BTBR* mice, which is in accordance with several previous experimental studies [55,59,60].

Our findings also showed that E159 effectively improved social deficits in *BTBR* mice, specifically enhancing sociability and social novelty behaviors. Improvements in sociability and social novelty were most significant with the 2.5 mg/kg dose of E159, while effects at higher doses (5 and 10 mg/kg) were less pronounced. These results align with a previous study where H3R antagonism decreased phencyclidine-induced social behavior impairments in animals [31]. Additionally, the reference drug ARP improved sociability and social novelty in *BTBR* mice, with effects comparable to those of E159. To account for the potential confounding effects of general locomotor activity and exploratory behavior on the outcomes of other behavioral studies, an open-field test was also carried out in *BTBR* and *B6* mice. *BTBR* mice consistently showed greater distances traveled compared to control mice. E159 did not impact movement in autistic mice. E159 treatment in control *B6* mice showed no adverse effects, indicating it does not alter baseline anxiety or exploratory behaviors. E159 also improved the time *BTBR* mice spent in the central arena, indicating it can modulate anxiety-associated fear levels, but it did not impact hyperactivity as it had no effect on the total distance traveled.

In *BTBR* mice, 2.5 mg/kg of E159 caused a significantly greater improvement in sociability, and spontaneous alternation, as well as reduced repetitive grooming behavior compared to higher doses of 5 and 10 mg/kg. This indicates that the optimal behavioral improvements occurred at 2.5 mg/kg, while higher doses may have caused off-target effects. These findings are also consistent with a previous study of E159 where significant improvement in memory in a DIZ-induced amnesia model, was obtained with 2.5 mg/kg of E159 compared to higher doses of 5 and 10 mg/kg [40]. Conversely, all doses of E159 had no impact on the behavior of *B6* animals, thereby ruling out any potential interfering effects of the treatment. Additionally, the improvement in behavioral impairment observed with E159 at 2.5 mg/kg was similar to those achieved with standard drug ARP. Therefore, an abrogation study using co-administration of the H3R agonist MHA was conducted with E159 at 2.5 mg/kg, as this was identified as the most optimal dose for minimizing any off-target effects that could occur with 5 or 10 mg/kg of E159. Notably, the co-administration of (*R*)-α-methylhistamine (MHA), an H3R agonist, nullified all beneficial effects on repetitive behavior, enhancement in alternation and social activity behavior exhibited by E159 (2.5 mg), strongly correlating the role of histaminergic neurotransmission in behavioral and cognitive improvements observed in *BTBR* mice following treatment with E159 (2.5 mg/kg, i.p.).

ASD patients often show motor coordination deficits and delayed motor skill development linked to cerebellar dysfunction. Temporal lobe abnormalities, particularly in the amygdala and hippocampus, also contribute to autism-like symptoms [3,61,62]. Immune dysregulation plays a critical role in ASD, with microglia activation leading to inflammation and immune dysfunction. Elevated pro-inflammatory cytokines are associated with ASD symptoms in both humans and *BTBR* mice. Children with ASD frequently exhibit abnormal immune responses, increased cytokine levels, and behavioral issues, such as learning deficits and stereotypical behaviors [11,63,64]. Excessive TNF-α production is a key factor in systemic inflammation and ASD development, suggesting that targeting inflammation could be a potential therapeutic approach for alleviating ASD symptoms [65,66].

Histamine itself can trigger the activation of microglia and inflammation, yet it has anti-inflammatory properties under stress-induced pathological conditions [67,68]. Several studies suggest that histamine interacts with its receptors on microglia and astrocytes, influencing their phenotypes and reducing neuroinflammation [69]. In a study, H3R antagonism was found to increase histamine release, activate H2 receptors, and trigger the PKA/cAMP/CREB pathway, reducing NF-κB/CBP interactions and shifting microglia from a pro-inflammatory M1 state to an anti-inflammatory M2 state, alleviating neuroinflammation [70]. Astrocytes, key regulators of brain homeostasis, can adopt pro-inflammatory (A1) or anti-inflammatory (A2) phenotypes. Thioperamide, an H3R antagonist, was shown to reduce inflammation by shifting astrocytes from A1 to A2 through CREB activation [58,70]. In our study, we observed a significant elevation of TNF-α, IL-6, and IL-1β in the cerebellum and hippocampus of *BTBR* mice. Consistent with the above-mentioned studies, H3R antagonist E159 (2.5 mg/kg) effectively reduced these proinflammatory markers, thereby inhibiting neuroinflammation in the brain. Moreover, ARP demonstrated a similar significant reduction in proinflammatory cytokines. Conversely, co-administration of E159 (2.5 mg) with the H3R agonist (*R*)-α-methylhistamine (MHA) increased pro-inflammatory cytokine levels, indicating that E159′s beneficial effects are mediated through interaction with central H3Rs, with brain histamine playing a role in its neuroprotective effects on ASD-like symptoms in *BTBR* mice (Table 2).

Neuronal autophagy plays a pivotal role in neuronal interaction, signaling, and development, and any disruption in this process can adversely impact memory formation, synaptic plasticity, and structural remodeling [71]. The appropriate axonal and dendritic growth is essential for maintaining neuronal equilibrium, with impaired organelles or proteins typically degraded to facilitate structural plasticity during development [72]. Consistent evidence underscores the significance of autophagy in dendritic, axonal, and synaptic development and maturation. Inhibition of autophagy disrupts the process leading to social behavior deficits and contributing to the development of ASD and other mental illnesses [73,74,75].

The cerebellum shows consistent abnormalities in individuals with ASD. Research suggests that in addition to motor coordination and balance, it plays a key role in cognitive functions such as executive function, working memory, and language—areas commonly impaired in those with ASD. This highlights the importance of cerebellar dysfunction in the condition [76]. Purkinje cells, the primary output cells of the cerebellum, play a key role in neurotransmission to the cortex. Research using animal models of ASD has demonstrated that dysfunction in Purkinje cells is linked to the behavioral abnormalities characteristic of ASD-like behaviors [77]. Early anatomical studies of postmortem ASD brain tissue revealed a significant reduction in Purkinje cell numbers in the lateral hemisphere. In cerebellar samples of individuals with ASD, abnormal activation of microglia and astrocytes, along with significant accumulation of monocytes and macrophages, is observed, particularly in the granular layer and white matter. These inflammatory changes are associated with notable histological abnormalities, including a reduction in Purkinje cells [77,78]. ASD patients also exhibit changes like abnormal cerebellar vermis size and overall cerebellar volume differences. Altered expression of GABA-related enzymes GAD65 and GAD67 in the cerebellum is well-documented in ASD. Reduced GAD65/67 levels in ASD impair inhibitory signaling, synaptic plasticity, and cerebellar computation, disrupting connections between the basket and Purkinje cells and affecting downstream targets like the deep cerebellar nuclei [79]. Functionally, impaired cerebellar development affects both motor and cognitive functions, with abnormalities linked to social interaction deficits [80]. The cerebellum is increasingly recognized as a key brain structure involved in the pathology of ASD; hence, we have focused on the cerebellum to explore the impact of E159 on autophagy within this region. Autophagy also acts as a protective mechanism against oxidative stress by regulating cellular ROS levels and removing damaged proteins and organelles [81,82]. It also suppresses the generation of inflammatory factors triggered by lipopolysaccharide by regulating innate immune signaling pathways and inflammasome activity. Earlier research indicates that activation of ROS and autophagy contribute to microglial activity [83]. Several upstream signals like PI3K-AKT, AMPK, and TSC1/2 regulate mTOR activity. A variety of genetic conditions are linked to ASD, including tuberous sclerosis complex, phosphatase and tensin homolog, hamartoma tumor syndrome, fragile X syndrome, and neurofibromatosis 1. Rodent models of these conditions have exhibited elevated mTORC1 activity in the brain, along with ASD-like behavioral deficits, which were reversed by treatment with the mTORC1 inhibitor rapamycin [84]. The AKT-mTOR pathway is an important signaling cascade associated with long-term plasticity and contributes to cognitive dysfunction. In addition to synaptic plasticity, mTOR has also been widely implicated in the inhibition of autophagy. Excessive activation of mTOR signaling relates to disruption in both glial and neuronal development, which is associated with the pathogenesis of ASD [85,86]. Several autophagy-related genes such as Beclin 1 are crucial in the process of autophagy [87]. Light chain3 (LC3) also serves as a significant indicator of autophagy [88]. A decrease in the levels of LC3 and Beclin1 leads to mitochondrial dysfunction and build-up of ROS, causing NLRP3 to generate IL-1β, escalating inflammation and neurotoxicity. This process ultimately results in neurodegeneration and neuronal death [89]. H3R activation can activate multiple intracellular signaling pathways, such as the PI3K/AKT pathway and mitogen-activated protein kinase (MAPK) pathway [90]. To further explore the impact of H3R antagonist E159, we analyzed the expression of autophagic markers, LC3A/B, and Beclin 1 in the cerebellum of *BTBR* mice. In this study, *BTBR* mice exhibited a reduction in the LC3A/B levels as well as decreased levels of Beclin1. These findings suggest impaired autophagy in the cerebellum. Also, cerebellar tissues from *BTBR* mice showed significantly elevated levels of p-mTOR/ mTOR compared to the *B6* control strain. However, treatment with E159 (2.5 mg/kg) significantly decreased p-mTOR levels in the cerebellum of E159-administered *BTBR* animals, which indicates the inhibition of mTOR activity. This is consistent with a previous preclinical study where H3R antagonism inhibits phosphorylation of mTOR and reinforces autophagy [36]. Wang et al. (2019) explored the effects of LC1405, a novel H3R antagonist, on cognitive deficits caused by Aβ in a mouse model of Alzheimer’s disease (APP/PS1). The study revealed that LC1405 effectively slowed disease progression by improving memory and learning, while also preventing neurodegeneration [91]. These effects were mediated through H3R modulation of cAMP/CREB and PI3K/AKT /GSK3β signaling. H3R blockade by E159 could potentially modulate the PI3K/AKT pathway leading to reduced mTOR activity and increased autophagy. Future studies are required to investigate whether E159 exerts its effects by modulating these upstream pathways, which could provide a more comprehensive understanding of its mechanistic role in both autophagy regulation and the treatment of autism. Notably, the inhibition of mTOR and consequent increase in the level of LC3A/B and Beclin 1 was reduced when the mice were treated with MHA (10 mg/kg) in addition to E159 (2.5 mg/kg), which further advocates the role of brain histamine in the protective actions of H3R antagonist E159. These preliminary findings indicate that H3R antagonist E159, which maintains crucial interactions in docking studies at H3R, could play a role in regulating the mTOR signaling pathway associated with autophagy and improve autism-like symptoms.

## 4. Materials and Methods

### 4.1. Molecular Docking Studies

Schrodinger 2022-4 was employed for docking purposes, with ligands prepared in their ionized forms (protonated N4 piperazine nitrogen, +1 charge) [92]. ConfGen was used to generate bioactive conformations (water environment at physiological pH, targeting 20 conformers per ligand) [93]. Only five conformers with the lowest energy were chosen for docking experiments. Docking was performed using the standard protocol on a rigid receptor with a ligand-centered grid (cubical box of A, extra precision) [94]. To validate the docking approach, native ligands were re-docked with high accuracy. Induced-fit docking was conducted with the Glide IGD module. The putative binding energy of ligands (dG) was calculated using Prime MM-GBSA [95].

Molecular dynamics simulations for generated complexes, conducted for 250 ns at 300 K (pressure 1.01325 bar, ensemble class NPγT), were carried out in Desmond [96] with calculated docking pose constituting the starting point for simulation. The protein’s membrane orientation was sourced from the OPM database [97]. The simulation extended for 250 ns, utilizing the TIP3P [98] solvent and POPC membrane model, and generated 1000 frames. Selected frames were analyzed using the Simulation Interaction Diagram tool and visual assessment. The figures shown were performed using the Maestro Schrodinger package.

### 4.2. In Vivo Studies

#### 4.2.1. Animals

Behavioral experiments were conducted using male *BTBR T+Itpr3tf/J (BTBR)* and *C57BL/6J (B6)* mice. At the start of the study, the *BTBR* mice were 8–10 weeks old and weighed between 27–32 g, and the *B6* mice were 8–10 weeks old and weighed between 22–28 g. The animals were housed in the CMHS Animal facility at UAE University with a 12 h light/dark cycle with regulated temperature and humidity and unrestricted access to food and water. All necessary measures were taken to ensure the ethical treatment of animals during our study. All procedures were conducted following approval from the Institutional Animal Ethics Committee of UAE University (Approval No. ERA-2017-5603). The behavioral studies were conducted between 8:00 a.m. and 3:00 p.m. To minimize animal suffering, we used the fewest number of animals to achieve our study objectives. Only male mice were selected for the study to reduce within-group variability caused by hormonal changes during the estrogenic cycle in female mice.

#### 4.2.2. Drug Compounds and Biochemical Materials

The test compound, E159 underwent development and in vitro pharmacological assessment at the Department of Technology and Biotechnology of Drugs (Kraków, Poland) [39]. E159 exhibits high and selective binding affinities to H3R compared to H4R and H1R. Aripiprazole (ARP, 1 mg/kg, i.p.), the reference drug, was procured from Sigma-Aldrich (St. Louis, MO, USA). Additionally, CNS-penetrant H3R agonist (*R*)-α-methylhistamine (MHA, 10 mg/kg) served for confirmatory studies following its sub-chronic systemic co-administration and was obtained from Sigma-Aldrich. The compounds were administered i.p. 30 min prior to the behavioral tests. Each animal received an injection adjusted to its body weight, with a volume of 10 mL/kg. Vehicle treatment consisted of 0.9% normal saline. The drug doses were selected according to prior studies [40,57]. Commercially available ELISA kits for proinflammatory cytokines (IL-1β, IL-6, and TNF-α) were procured from R&D Systems (Minneapolis, MN, USA). Bovine serum albumin (BSA), primary antibodies, and secondary antibodies were purchased from Cell Signaling, Danvers, MA, USA. PVDF membrane was sourced from Bio-Rad Laboratories (Hercules, CA, USA). The Pierce™ BCA Protein Assay Kit and Chemiluminescence Pico Kit were obtained from Thermo Fisher Scientific (Rockford, IL, USA).

#### 4.2.3. Study Design and Treatment

Prior to the experiment, all mice were acclimated for a week. The treatment lasted for 21 days in a sub-chronic regimen. *B6* mice used as the control group (group 1, n = 6) received VEH. *BTBR* mice injected with VEH (group 2, n = 6) functioned as the control group for ASD-related features. *BTBR* mice were administered with varying doses of E159 (2.5, 5 and 10 mg/kg, i.p.) (groups 3–5 respectively, n = 6). As the reference compound, Aripiprazole (ARP) was administered at a dosage of 1 mg/kg to *BTBR* mice (group 6, n = 6). For the abrogation studies, E159 (2.5 mg/kg) was co-administered with (*R*)-α-methylhistamine (MHA, 10 mg/kg, i.p.) (group 7) in *BTBR* mice. Besides the previously described groups, four additional groups of *B6* mice (n = 6) were treated with E159 (2.5–10 mg/kg) and ARP to control for any potential confounding effects of these treatments on the behaviors of the control *B6* mice (Table 1). The sub-chronic treatment (given i.p.) started a week prior to behavior experiments and continued until the sacrifice. On the last day of systemic treatment, after completing all behavioral tests, the animals were sacrificed. Their skulls were opened, and the brains were extracted. The hemispheres were separated, and the cerebellum and hippocampus were isolated and promptly frozen in liquid nitrogen for future biochemical analysis.

#### 4.2.4. Behavioral Assessments

##### Self-Grooming

The assessment of self-grooming was carried out as described in previous studies [99,100]. Following a 10 min habituation period, the duration of grooming was measured for the second 10 min testing phase.

##### Y-Maze or Spontaneous Alteration

The test assesses rodents’ exploratory behavior when introduced to a novel environment [57,101]. It also tests the working memory function in mice [56]. The Y-maze test began with mice being placed into one arm of a Y-shaped maze. The test duration was 8 min. Three consecutive, non-repeating arm entries were considered correct spontaneous alternations (SAB). Over an 8 min period, the total movements made by the mice, as well as the percentage of alternations were then analyzed.

##### Three Chamber Social Test (TCT)

A TCT assessed sociability and social novelty in mice in accordance with previous reports [32,35,102]. The experiment used a three-chambered apparatus with a central chamber and two side chambers accessible through square doors. The test comprised a 30 min duration: a habituation phase, followed by sessions with novel and familiar mice placed in separate chambers. Social behavior was assessed using the Sociability Index (SI) and Social Novelty Index (SNI), based on the duration of exploring novel versus familiar mice.

##### Open Field Test (OFT)

The OFT was employed to assess the impact of treatments on both anxiety-like behaviors and locomotor activity in tested animals [32,103,104]. Briefly, the test began with a 5 min acclimatization period. The study measured both the total distance traveled throughout the arena and the duration spent in the central versus peripheral zones for ten min. Reduced time spent in the central area suggested anxiety-like behavior, whereas the total distance traveled reflected the animals’ overall locomotor activity [105].

### 4.3. Biochemical Investigations

#### 4.3.1. Brain Collection and Tissue Processing

The cerebellum and hippocampus were stored at −80 °C for future experiments [35]. RIPA buffer consisting of protease inhibitors and phosphatase inhibitors was used to homogenize the tissues, followed by centrifugation at 12,000 rpm (4 °C, 30 min) for removal of tissue debris. The supernatants were separated and used later for the estimation of proinflammatory cytokines and Western blot analysis.

#### 4.3.2. Pro-Inflammatory Cytokine Assessments

ELISA was used to quantify TNF-α, IL-1β, and IL-6 in the cerebellum and hippocampus, following the manufacturer’s guidelines [106,107].

#### 4.3.3. Western Blot

The cerebellum homogenates were analyzed for protein concentration based on earlier reports [107]. Proteins separated on 12% gel were transferred to a PVDF membrane pre-activated with methanol using 90 V for 1 h 30 min. The membranes after blocking with 5% BSA (1 h, 4 °C) were incubated overnight with monoclonal antibodies against Actin, mTOR, p-mTOR, LC3A/B (all at 1:1000 dilution, Cell Signaling Technologies, Danvers, MA, USA), and Beclin1 (1:1000, Santa Cruz, CA, USA) at 4 °C. The following day after washing with TBST, the membranes were incubated with HRP-conjugated secondary antibodies for 3 h at 4 °C. The Super Signal West Pico PLUS Chemiluminescent Substrate (Thermo Scientific, Rockford, IL, USA) was used to visualize the protein bands and quantified with ImageJ software (Version 1.8.0) (NIH).

### 4.4. Statistical Analyses

Data were presented as mean ± SEM. A two-way ANOVA combined with Tukey’s post hoc test was employed to examine the effects of drug treatment. For proinflammatory cytokines and protein expressions, one-way ANOVA was used. Statistical analyses were conducted with GraphPad Prism (Version 8.0), and *p*-values below 0.05 denoted statistical significance (*p* < 0.05).

## 5. Conclusions

Brain histamine influences numerous functions, such as memory, long-term social recognition, cognition, learning, and emotions. In the current series of investigations, we observed that H3R antagonist E159 effectively alleviated ASD-related behaviors in *BTBR* mouse model of autism, specifically enhancing sociability and reducing repetitive behaviors, which are core symptoms of ASD. These improvements can be attributed to E159′s ability to modulate histaminergic neurotransmission as MHA, a centrally acting H3R agonist reversed these E159-provided effects. These behavioral improvements were observed simultaneously with a significant reduction of neuroinflammation and profound mitigation of deficits in autophagy. Our preliminary observations suggest that targeting H3Rs with antagonists like E159 could be a promising therapeutic approach for alleviating ASD-related symptoms.

### Limitation

While we demonstrate that E159 ameliorates autism-like behaviors and enhances autophagy through modulation of the mTOR pathway, we did not extensively investigate the upstream signaling factors or related pathways influencing the regulation of autophagy. Secondly, our study focused on the effects of sub-chronic E159 treatment in *BTBR* mice, and we did not evaluate its long-term impact. This limits our understanding of the compound’s potential chronic effects, which warrants further investigation. Lastly, we did not evaluate the activation or inactivation of specific cerebellar cell types following treatment with test compound E159. Understanding the cell-specific functional responses of H3Rs would offer valuable insights into its modulating role in the cerebellum. Future studies employing techniques such as immunohistochemistry and electrophysiology will be necessary to address these gaps.

## Figures and Tables

**Table 1:** Behavioral outcomes in control *B6* mice following systemic pretreatment with E159 and ARP.

Behavioral Test		VEH	E159 (mg/kg, i.p.)			ARP (1 mg/kg, i.p.)
			2.5	5	10	
Self-grooming (s)		58.5 ± 2.89	57.5 ± 1.71	57.5 ± 2.17	55 ± 3.15	59.33 ± 3.93
Spontaneous alteration (%)		76.75 ± 3.1	73.99 ± 3.78	72.54 ± 1.98	74.58 ± 1.55	78.69 ± 0.54
Open Field	Time in center (s)	59.5 ± 4.94	56.93 ± 4.42	57.83 ± 2.81	54.5 ± 6.69	62.5 ± 2.43
	Time in periphery (s)	538.8 ± 4.5	543 ± 4.36	542.2 ± 2.81	545.5 ± 6.69	537.5 ± 2.43
	Total distance travelled (cm)	2691 ± 203.6	2584 ± 219.5	2545 ± 213	2505 ± 215.5	2730 ± 95.66
Three Chamber Test	Sociability Index (SI)	0.42 ± 0.07	0.41 ± 0.05	0.40 ± 0.03	0.39 ± 0.08	0.43 ± 0.05
	Social Novelty Index (SNI)	0.38 ± 0.05	0.36 ± 0.09	0.37 ± 0.07	0.37 ± 0.04	0.35 ± 0.07

Data are summarized as mean ± SEM (n = 6). No considerable differences were identified among VEH (Vehicle), E159, or ARP administered *B6* control mice.

**Table 2:** E159 mitigated neuroinflammation in cerebellum and hippocampus of *BTBR* mice.

Treatment Groups	Cerebellum			Hippocampus		
	Proinflammatory Cytokines			Proinflammatory Cytokines		
	TNF-α	IL-6	IL-1β	TNF-α	IL-6	IL-1β
*B6* (Ctrl) (VEH)	203.8 ± 4.9	67 ± 6.29	161.5 ± 3.13	191.4 ± 9.41	51.42 ± 1.9	70.66 ± 12.1
*BTBR* (Ctrl) (VEH)	275.7 ± 13.86 *	118.1 ± 8.58 *	340.9 ± 8.702 *	259.9 ± 10.11 *	95.08 ± 5.31 *	166.2 ± 11.36 *
*BTBR* (E159, 2.5 mg/kg)	224.6 ± 5.03 ^###^	72.26 ± 5.86 **	198.3 ± 10.5 ^##^	207.0 ± 11.81 ^##^	58.52 ± 2.18 **	96.73 ± 7.93 ^##^
*BTBR* (ARP, 1 mg/kg)	229.4 ± 6.5 ^###^	77.54 ± 3.84 ^##^	207.7 ± 10.84 ^##^	199.8 ± 3.45 ***	59.69 ± 4.83 ***	94.80 ± 15.51 ^##^
*BTBR* (E159, 2.5 mg/kg) + MHA	268.6 ± 15.49 ^$^	100.3 ± 8.28 ^$^	273.5 ± 15.29 ^$$^	244.8 ± 6.99 ^$^	90.46 ± 9.29 ^$$^	147.2 ± 6.48 ^$^

Tumor Necrosis Factor (TNF-α, pg/mg protein) and interleukin (IL-1β and IL-6, pg/mg protein) were assessed. *BTBR* mice exhibited considerable elevation in tested cytokine levels in cerebellum and hippocampus compared to control mice. E159 or ARP were administered for 21 days in *BTBR* mouse model. E159 considerably decreased cytokine levels in cerebellum and hippocampus. The modulation of proinflammatory cytokines by E159 (2.5 mg) was evaluated following a chronic (21-day) co-treatment with MHA. * *p* < 0.001 vs. *B6* mice treated with vehicle. ** *p* < 0.001, *** *p* < 0.01, ^##^ *p* < 0.01, ^###^ *p* < 0.05 vs. *BTBR* mice treated with vehicle. ^$^ *p* < 0.05, ^$$^ *p* < 0.01 vs. *BTBR* mice treated with E159 (2.5 mg), (mean ± SEM, n = 6).

## Data Availability

Data is contained within the article.

## References

[1] Thomas S.D., Jha N.K., Ojha S., Sadek B. (2023). mTOR Signaling Disruption and Its Association with the Development of Autism Spectrum Disorder. Molecules.

[2] Kumar S., Reynolds K., Ji Y., Gu R., Rai S., Zhou C.J. (2019). Impaired Neurodevelopmental Pathways in Autism Spectrum Disorder: A Review of Signaling Mechanisms and Crosstalk. J. Neurodev. Disord..

[3] Dana H., Tahtasakal R., Sener E.F. (2020). Animal Models of Autism: A Perspective from Autophagy Mechanisms. J. Transl. Genet. Genom..

[4] Jiang P., Zhou L., Zhao L., Fei X., Wang Z., Liu T., Tang Y., Li D., Gong H., Luo Y. (2024). Puerarin Attenuates Valproate-Induced Features of ASD in Male Mice via Regulating Slc7a11-Dependent Ferroptosis. Neuropsychopharmacology.

[5] Bjorklund G., Saad K., Chirumbolo S., Kern J.K., Geier D.A., Geier M.R., Urbina M.A. (2016). Immune Dysfunction and Neuroinflammation in Autism Spectrum Disorder. Acta Neurobiol. Exp..

[6] Siniscalco D., Schultz S., Brigida A.L., Antonucci N. (2018). Inflammation and Neuro-Immune Dysregulations in Autism Spectrum Disorders. Pharmaceuticals.

[7] Pangrazzi L., Balasco L., Bozzi Y. (2020). Oxidative Stress and Immune System Dysfunction in Autism Spectrum Disorders. Int. J. Mol. Sci..

[8] Jiang C.-C., Lin L.-S., Long S., Ke X.-Y., Fukunaga K., Lu Y.-M., Han F. (2022). Signalling Pathways in Autism Spectrum Disorder: Mechanisms and Therapeutic Implications. Signal Transduct. Target. Ther..

[9] Eftekharian M.M., Ghafouri-Fard S., Noroozi R., Omrani M.D., Arsang-jang S., Ganji M., Gharzi V., Noroozi H., Komaki A., Mazdeh M. (2018). Cytokine Profile in Autistic Patients. Cytokine.

[10] Usui N., Kobayashi H., Shimada S. (2023). Neuroinflammation and Oxidative Stress in the Pathogenesis of Autism Spectrum Disorder. Int. J. Mol. Sci..

[11] Vargas D.L., Nascimbene C., Krishnan C., Zimmerman A.W., Pardo C.A. (2005). Neuroglial Activation and Neuroinflammation in the Brain of Patients with Autism. Ann. Neurol..

[12] Lee G.A., Lin Y.-K., Lai J.-H., Lo Y.-C., Yang Y.-C.S.H., Ye S.-Y., Lee C.-J., Wang C.-C., Chiang Y.-H., Tseng S.-H. (2021). Maternal Immune Activation Causes Social Behavior Deficits and Hypomyelination in Male Rat Offspring with an Autism-Like Microbiota Profile. Brain Sci..

[13] Onore C., Yang H., Van de Water J., Ashwood P. (2017). Dynamic Akt/mTOR Signaling in Children with Autism Spectrum Disorder. Front. Pediatr..

[14] Cui Y., Yang M., Wang Y., Ren J., Lin P., Cui C., Song J., He Q., Hu H., Wang K. (2021). Melatonin Prevents Diabetes-Associated Cognitive Dysfunction from Microglia-Mediated Neuroinflammation by Activating Autophagy via TLR4/Akt/mTOR Pathway. FASEB J..

[15] Han H.-E., Kim T.-K., Son H.-J., Park W.J., Han P.-L. (2013). Activation of Autophagy Pathway Suppresses the Expression of iNOS, IL6 and Cell Death of LPS-Stimulated Microglia Cells. Biomol. Ther..

[16] Wang K. (2015). Autophagy and Apoptosis in Liver Injury. Cell Cycle.

[17] Matsuzawa-Ishimoto Y., Hwang S., Cadwell K. (2018). Autophagy and Inflammation. Annu. Rev. Immunol..

[18] Deng Z., Zhou X., Lu J.-H., Yue Z. (2021). Autophagy Deficiency in Neurodevelopmental Disorders. Cell Biosci..

[19] Lieberman O.J., Cartocci V., Pigulevskiy I., Molinari M., Carbonell J., Broseta M.B., Post M.R., Sulzer D., Borgkvist A., Santini E. (2020). mTOR Suppresses Macroautophagy During Striatal Postnatal Development and Is Hyperactive in Mouse Models of Autism Spectrum Disorders. Front. Cell. Neurosci..

[20] Huber K.M., Klann E., Costa-Mattioli M., Zukin R.S. (2015). Dysregulation of Mammalian Target of Rapamycin Signaling in Mouse Models of Autism. J. Neurosci..

[21] Agam G., Taylor Z., Vainer E., Golan H.M. (2020). The Influence of Choline Treatment on Behavioral and Neurochemical Autistic-like Phenotype in Mthfr-Deficient Mice. Transl. Psychiatry.

[22] Eissa N., Sadeq A., Sasse A., Sadek B. (2020). Role of Neuroinflammation in Autism Spectrum Disorder and the Emergence of Brain Histaminergic System. Lessons Also for BPSD?. Front. Pharmacol..

[23] Baronio D., Castro K., Gonchoroski T., de Melo G.M., Nunes G.D.F., Bambini-Junior V., Gottfried C., Riesgo R. (2015). Effects of an H3R Antagonist on the Animal Model of Autism Induced by Prenatal Exposure to Valproic Acid. PLoS ONE.

[24] Wright C., Shin J.H., Rajpurohit A., Deep-Soboslay A., Collado-Torres L., Brandon N.J., Hyde T.M., Kleinman J.E., Jaffe A.E., Cross A.J. (2017). Altered Expression of Histamine Signaling Genes in Autism Spectrum Disorder. Transl. Psychiatry.

[25] Eissa N., Azimullah S., Jayaprakash P., Jayaraj R.L., Reiner D., Ojha S.K., Beiram R., Stark H., Łażewska D., Kieć-Kononowicz K. (2019). The Dual-Active Histamine H3 Receptor Antagonist and Acetylcholine Esterase Inhibitor E100 Ameliorates Stereotyped Repetitive Behavior and Neuroinflammmation in Sodium Valproate Induced Autism in Mice. Chem. Biol. Interact..

[26] Sadek B., Saad A., Sadeq A., Jalal F., Stark H. (2016). Histamine H3 Receptor as a Potential Target for Cognitive Symptoms in Neuropsychiatric Diseases. Behav. Brain Res..

[27] Guilloux J.-P., Samuels B.A., Mendez-David I., Hu A., Levinstein M., Faye C., Mekiri M., Mocaer E., Gardier A.M., Hen R. (2017). S 38093, a Histamine H3 Antagonist/Inverse Agonist, Promotes Hippocampal Neurogenesis and Improves Context Discrimination Task in Aged Mice. Sci. Rep..

[28] Eissa N., Khan N., Ojha S.K., Łazewska D., Kieć-Kononowicz K., Sadek B. (2018). The Histamine H3 Receptor Antagonist DL77 Ameliorates MK801-Induced Memory Deficits in Rats. Front. Neurosci..

[29] Brown J.W., Whitehead C.A., Basso A.M., Rueter L.E., Zhang M. (2013). Preclinical Evaluation of Non-Imidazole Histamine H3 Receptor Antagonists in Comparison to Atypical Antipsychotics for the Treatment of Cognitive Deficits Associated with Schizophrenia. Int. J. Neuropsychopharmacol..

[30] Zhang M., Jiao J., Hu X., Yang P., Huang Y., Situ M., Guo K., Cai J., Huang Y. (2020). Exploring the Spatial Working Memory and Visual Perception in Children with Autism Spectrum Disorder and General Population with High Autism-like Traits. PLoS ONE.

[31] Griebel G., Pichat P., Pruniaux M.-P., Beeské S., Lopez-Grancha M., Genet E., Terranova J.-P., Castro A., Sánchez J.A., Black M. (2012). SAR110894, a Potent Histamine H_3_-Receptor Antagonist, Displays Procognitive Effects in Rodents. Pharmacol. Biochem. Behav..

[32] Eissa N., Jayaprakash P., Azimullah S., Ojha S.K., Al-Houqani M., Jalal F.Y., Łażewska D., Kieć-Kononowicz K., Sadek B. (2018). The Histamine H3R Antagonist DL77 Attenuates Autistic Behaviors in a Prenatal Valproic Acid-Induced Mouse Model of Autism. Sci. Rep..

[33] Eissa N., Azimullah S., Jayaprakash P., Jayaraj R.L., Reiner D., Ojha S.K., Beiram R., Stark H., Łażewska D., Kieć-Kononowicz K. (2020). The Dual-Active Histamine H3 Receptor Antagonist and Acetylcholine Esterase Inhibitor E100 Alleviates Autistic-Like Behaviors and Oxidative Stress in Valproic Acid Induced Autism in Mice. Int. J. Mol. Sci..

[34] Thomas S.D., Abdalla S., Eissa N., Akour A., Jha N.K., Ojha S., Sadek B. (2024). Targeting Microglia in Neuroinflammation: H3 Receptor Antagonists as a Novel Therapeutic Approach for Alzheimer’s Disease, Parkinson’s Disease, and Autism Spectrum Disorder. Pharmaceuticals.

[35] Venkatachalam K., Eissa N., Awad M.A., Jayaprakash P., Zhong S., Stölting F., Stark H., Sadek B. (2021). The Histamine H3R and Dopamine D2R/D3R Antagonist ST-713 Ameliorates Autism-like Behavioral Features in *BTBR* T+tf/J Mice by Multiple Actions. Biomed. Pharmacother..

[36] Yan H., Zhang X., Hu W., Ma J., Hou W., Zhang X., Wang X., Gao J., Shen Y., Lv J. (2014). Histamine H3 Receptors Aggravate Cerebral Ischaemic Injury by Histamine-Independent Mechanisms. Nat. Commun..

[37] Meyza K.Z., Blanchard D.C. (2017). The *BTBR* Mouse Model of Idiopathic Autism—Current View on Mechanisms. Neurosci. Biobehav. Rev..

[38] Wu H., Zhao G., Liu S., Zhang Q., Wang P., Cao Y., Wu L. (2022). Supplementation with Selenium Attenuates Autism-like Behaviors and Improves Oxidative Stress, Inflammation and Related Gene Expression in an Autism Disease Model. J. Nutr. Biochem..

[39] Łażewska D., Ligneau X., Schwartz J.-C., Schunack W., Stark H., Kieć-Kononowicz K. (2006). Ether Derivatives of 3-Piperidinopropan-1-Ol as Non-Imidazole Histamine H3 Receptor Antagonists. Bioorg. Med. Chem..

[40] Alachkar A., Łażewska D., Kieć-Kononowicz K., Sadek B. (2017). The Histamine H3 Receptor Antagonist E159 Reverses Memory Deficits Induced by Dizocilpine in Passive Avoidance and Novel Object Recognition Paradigm in Rats. Front. Pharmacol..

[41] McFarlane H.G., Kusek G.K., Yang M., Phoenix J.L., Bolivar V.J., Crawley J.N. (2008). Autism-like Behavioral Phenotypes in *BTBR* T+tf/J Mice. Genes Brain Behav..

[42] Amodeo D.A., Jones J.H., Sweeney J.A., Ragozzino M.E. (2012). Differences in *BTBR T+ Tf/J* and *C57BL/6J* Mice on Probabilistic Reversal Learning and Stereotyped Behaviors. Behav. Brain Res..

[43] Shimamura T., Shiroishi M., Weyand S., Tsujimoto H., Winter G., Katritch V., Abagyan R., Cherezov V., Liu W., Han G.W. (2011). Structure of the Human Histamine H1 Receptor Complex with Doxepin. Nature.

[44] Peng X., Yang L., Liu Z., Lou S., Mei S., Li M., Chen Z., Zhang H. (2022). Structural Basis for Recognition of Antihistamine Drug by Human Histamine Receptor. Nat. Commun..

[45] Im D., Kishikawa J., Shiimura Y., Hisano H., Ito A., Fujita-Fujiharu Y., Sugita Y., Noda T., Kato T., Asada H. (2023). Structural Insights into the Agonists Binding and Receptor Selectivity of Human Histamine H4 Receptor. Nat. Commun..

[46] Jończyk J., Malawska B., Bajda M. (2017). Hybrid Approach to Structure Modeling of the Histamine H3 Receptor: Multi-Level Assessment as a Tool for Model Verification. PLoS ONE.

[47] Kas M.J., Glennon J.C., Buitelaar J., Ey E., Biemans B., Crawley J., Ring R.H., Lajonchere C., Esclassan F., Talpos J. (2014). Assessing Behavioural and Cognitive Domains of Autism Spectrum Disorders in Rodents: Current Status and Future Perspectives. Psychopharmacology.

[48] Karagiannidis I., Dehning S., Sandor P., Tarnok Z., Rizzo R., Wolanczyk T., Madruga-Garrido M., Hebebrand J., Nöthen M.M., Lehmkuhl G. (2013). Support of the Histaminergic Hypothesis in Tourette Syndrome: Association of the Histamine Decarboxylase Gene in a Large Sample of Families. J. Med. Genet..

[49] Rapanelli M., Frick L., Pogorelov V., Ohtsu H., Bito H., Pittenger C. (2017). Histamine H3R Receptor Activation in the Dorsal Striatum Triggers Stereotypies in a Mouse Model of Tic Disorders. Transl. Psychiatry.

[50] Estes M.L., McAllister A.K. (2015). Immune Mediators in the Brain and Peripheral Tissues in Autism Spectrum Disorder. Nat. Rev. Neurosci..

[51] Onore C.E., Careaga M., Babineau B.A., Schwartzer J.J., Berman R.F., Ashwood P. (2013). Inflammatory Macrophage Phenotype in *BTBR* T+tf/J Mice. Front. Neurosci..

[52] Ahmad S.F., Ansari M.A., Nadeem A., Bakheet S.A., Alshammari M.A., Khan M.R., Alsaad A.M.S., Attia S.M. (2018). S3I-201, a Selective Stat3 Inhibitor, Restores Neuroimmune Function through Upregulation of Treg Signaling in Autistic *BTBR T+ Itpr3tf/J* Mice. Cell. Signal..

[53] McTighe S.M., Neal S.J., Lin Q., Hughes Z.A., Smith D.G. (2013). The *BTBR* Mouse Model of Autism Spectrum Disorders Has Learning and Attentional Impairments and Alterations in Acetylcholine and Kynurenic Acid in Prefrontal Cortex. PLoS ONE.

[54] Silverman J.L., Tolu S.S., Barkan C.L., Crawley J.N. (2010). Repetitive Self-Grooming Behavior in the *BTBR* Mouse Model of Autism Is Blocked by the mGluR5 Antagonist MPEP. Neuropsychopharmacology.

[55] Choi H.J., Im S.J., Park H.R., Park S., Kim C.-E., Ryu S. (2019). Long-Term Effects of Aripiprazole Treatment during Adolescence on Cognitive Function and Dopamine D2 Receptor Expression in Neurodevelopmentally Normal Rats. Clin. Psychopharmacol. Neurosci..

[56] Kraeuter A.-K., Guest P.C., Sarnyai Z. (2019). The Y-Maze for Assessment of Spatial Working and Reference Memory in Mice. Methods Mol. Biol..

[57] Eissa N., Venkatachalam K., Jayaprakash P., Falkenstein M., Dubiel M., Frank A., Reiner-Link D., Stark H., Sadek B. (2021). The Multi-Targeting Ligand ST-2223 with Histamine H3 Receptor and Dopamine D2/D3 Receptor Antagonist Properties Mitigates Autism-Like Repetitive Behaviors and Brain Oxidative Stress in Mice. Int. J. Mol. Sci..

[58] Wang J., Liu B., Xu Y., Luan H., Wang C., Yang M., Zhao R., Song M., Liu J., Sun L. (2022). Thioperamide Attenuates Neuroinflammation and Cognitive Impairments in Alzheimer’s Disease via Inhibiting Gliosis. Exp. Neurol..

[59] Shin C.Y., Kim H.-S., Cha K.-H., Won D.H., Lee J.-Y., Jang S.W., Sohn U.D. (2018). The Effects of Donepezil, an Acetylcholinesterase Inhibitor, on Impaired Learning and Memory in Rodents. Biomol. Ther..

[60] Lamothe H., Schreiweis C., Mondragón-González L.S., Rebbah S., Lavielle O., Mallet L., Burguière E. (2023). The Sapap3^−/−^ Mouse Reconsidered as a Comorbid Model Expressing a Spectrum of Pathological Repetitive Behaviours. Transl. Psychiatry.

[61] Mostofsky S.H., Goldberg M.C., Landa R.J., Denckla M.B. (2000). Evidence for a Deficit in Procedural Learning in Children and Adolescents with Autism: Implications for Cerebellar Contribution. J. Int. Neuropsychol. Soc..

[62] Marko M.K., Crocetti D., Hulst T., Donchin O., Shadmehr R., Mostofsky S.H. (2015). Behavioural and Neural Basis of Anomalous Motor Learning in Children with Autism. Brain.

[63] Ashwood P., Krakowiak P., Hertz-Picciotto I., Hansen R., Pessah I., Van de Water J. (2011). Elevated Plasma Cytokines in Autism Spectrum Disorders Provide Evidence of Immune Dysfunction and Are Associated with Impaired Behavioral Outcome. Brain Behav. Immun..

[64] Matta S.M., Hill-Yardin E.L., Crack P.J. (2019). The Influence of Neuroinflammation in Autism Spectrum Disorder. Brain Behav. Immun..

[65] Li X., Chauhan A., Sheikh A.M., Patil S., Chauhan V., Li X.-M., Ji L., Brown T., Malik M. (2009). Elevated Immune Response in the Brain of Autistic Patients. J. Neuroimmunol..

[66] Goines P.E., Ashwood P. (2013). Cytokine Dysregulation in Autism Spectrum Disorders (ASD): Possible Role of the Environment. Neurotoxicol. Teratol..

[67] Barata-Antunes S., Cristóvão A.C., Pires J., Rocha S.M., Bernardino L. (2017). Dual Role of Hista mine on Microglia-Induced Neurodegeneration. Biochim. Biophys. Acta (BBA)-Mol. Basis Dis..

[68] Saraiva C., Barata-Antunes S., Santos T., Ferreiro E., Cristóvão A.C., Serra-Almeida C., Ferreira R., Bernardino L. (2019). Histamine Modulates Hippocampal Inflammation and Neurogenesis in Adult Mice. Sci. Rep..

[69] Zhou Z., An Q., Zhang W., Li Y., Zhang Q., Yan H. (2024). Histamine and Receptors in Neuroinflammation: Their Roles on Neurodegenerative Diseases. Behav. Brain Res..

[70] Wang J., Liu B., Sun F., Xu Y., Luan H., Yang M., Wang C., Zhang T., Zhou Z., Yan H. (2022). Histamine H3R Antagonist Counteracts the Impaired Hippocampal Neurogenesis in Lipopolysaccharide-Induced Neuroinflammation. Int. Immunopharmacol..

[71] Rubinsztein D.C., Codogno P., Levine B. (2012). Autophagy Modulation as a Potential Therapeutic Target for Diverse Diseases. Nat. Rev. Drug Discov..

[72] Nixon R.A., Cataldo A.M. (2006). Lysosomal System Pathways: Genes to Neurodegeneration in Alzheimer’s Disease. J. Alzheimers Dis..

[73] Bowling H., Klann E. (2014). Shaping Dendritic Spines in Autism Spectrum Disorder: mTORC1-Dependent Macroautophagy. Neuron.

[74] Tang G., Gudsnuk K., Kuo S.-H., Cotrina M.L., Rosoklija G., Sosunov A., Sonders M.S., Kanter E., Castagna C., Yamamoto A. (2014). Loss of mTOR-Dependent Macroautophagy Causes Autistic-like Synaptic Pruning Deficits. Neuron.

[75] Zhang J., Zhang J.-X., Zhang Q.-L. (2016). PI3K/AKT/mTOR-Mediated Autophagy in the Development of Autism Spectrum Disorder. Brain Res. Bull..

[76] Biswas M.S., Roy S.K., Hasan R., Pk M.M.U. (2024). The Crucial Role of the Cerebellum in Autism Spectrum Disorder: Neuroimaging, Neurobiological, and Anatomical Insights. Health Sci. Rep..

[77] Zhou L., Jiang P., Zhao L., Fei X., Tang Y., Luo Y., Gong H., Wang X., Li X., Li S. (2024). Ligustilide Inhibits Purkinje Cell Ferritinophagy via the ULK1/NCOA4 Pathway to Attenuate Valproic Acid-Induced Autistic Features. Phytomedicine.

[78] Mapelli L., Soda T., D’Angelo E., Prestori F. (2022). The Cerebellar Involvement in Autism Spectrum Disorders: From the Social Brain to Mouse Models. Int. J. Mol. Sci..

[79] Sydnor L.M., Aldinger K.A. (2022). Structure, Function, and Genetics of the Cerebellum in Autism. J. Psychiatry Brain Sci..

[80] Su L.-D., Xu F.-X., Wang X.-T., Cai X.-Y., Shen Y. (2021). Cerebellar Dysfunction, Cerebro-Cerebellar Connectivity and Autism Spectrum Disorders. Neuroscience.

[81] Giordano S., Darley-Usmar V., Zhang J. (2014). Autophagy as an Essential Cellular Antioxidant Pathway in Neurodegenerative Disease. Redox Biol..

[82] Menezo Y.J.R., Elder K., Dale B. (2015). Link Between Increased Prevalence of Autism Spectrum Disorder Syndromes and Oxidative Stress, DNA Methylation, and Imprinting: The Impact of the Environment. JAMA Pediatr..

[83] He Y., She H., Zhang T., Xu H., Cheng L., Yepes M., Zhao Y., Mao Z. (2018). P38 MAPK Inhibits Autophagy and Promotes Microglial Inflammatory Responses by Phosphorylating ULK1. J. Cell Biol..

[84] Sato A., Ikeda K. (2022). Genetic and Environmental Contributions to Autism Spectrum Disorder Through Mechanistic Target of Rapamycin. Biol. Psychiatry Glob. Open Sci..

[85] Kaeberlein M. (2013). mTOR Inhibition: From Aging to Autism and Beyond. Scientifica.

[86] Takei N., Nawa H. (2014). mTOR Signaling and Its Roles in Normal and Abnormal Brain Development. Front. Mol. Neurosci..

[87] Kang R., Zeh H.J., Lotze M.T., Tang D. (2011). The Beclin 1 Network Regulates Autophagy and Apoptosis. Cell Death Differ..

[88] Qin L., Dai X., Yin Y. (2016). Valproic Acid Exposure Sequentially Activates Wnt and mTOR Pathways in Rats. Mol. Cell. Neurosci..

[89] Chen X., Zhou X., Cheng X., Lin L., Wang Q., Zhan R., Wu Q., Liu S. (2023). Protective Effect of Ferulic Acid on Lipopolysaccharide-Induced BV2 Microglia Inflammation via AMPK/mTOR Signaling Pathway. Molecules.

[90] Bongers G., Bakker R.A., Leurs R. (2007). Molecular Aspects of the Histamine H3 Receptor. Biochem. Pharmacol..

[91] Wang L., Fang J., Jiang H., Wang Q., Xue S., Li Z., Liu R. (2019). 7-Pyrrolidinethoxy-4′-Methoxyisoflavone Prevents Amyloid β–Induced Injury by Regulating Histamine H3 Receptor-Mediated cAMP/CREB and AKT/GSK3β Pathways. Front. Neurosci..

[92] Schrödinger (2017). Release 2022-4: Schrödinger Suite 2022-4.

[93] Watts K.S., Dalal P., Murphy R.B., Sherman W., Friesner R.A., Shelley J.C. (2010). ConfGen: A Conformational Search Method for Efficient Generation of Bioactive Conformers. J. Chem. Inf. Model..

[94] Friesner R.A., Banks J.L., Murphy R.B., Halgren T.A., Klicic J.J., Mainz D.T., Repasky M.P., Knoll E.H., Shelley M., Perry J.K. (2004). Glide: A New Approach for Rapid, Accurate Docking and Scoring. 1. Method and Assessment of Docking Accuracy. J. Med. Chem..

[95] Jacobson M.P., Pincus D.L., Rapp C.S., Day T.J.F., Honig B., Shaw D.E., Friesner R.A. (2004). A Hierarchical Approach to All-Atom Protein Loop Prediction. Proteins Struct. Funct. Genet..

[96] Bowers K.J., Chow E., Xu H., Dror R.O., Eastwood M.P., Gregersen B.A., Klepeis J.L., Kolossvary I., Moraes M.A., Sacerdoti F.D. Scalable Algorithms for Molecular Dynamics Simulations on Commodity Clusters. Proceedings of the 2006 ACM/IEEE Conference on Supercomputing (SC’06).

[97] Lomize M.A., Lomize A.L., Pogozheva I.D., Mosberg H.I. (2006). OPM: Orientations of Proteins in Membranes Database. Bioinformatics.

[98] Jorgensen W.L., Chandrasekhar J., Madura J.D., Impey R.W., Klein M.L. (1983). Comparison of Simple Potential Functions for Simulating Liquid Water. J. Chem. Phys..

[99] Cai Y., Wang L., Xiao R., Li X., He X., Gao J., Xu H., Fan X. (2017). Autism-like Behavior in the *BTBR* Mouse Model of Autism Is Improved by Propofol. Neuropharmacology.

[100] Sun J., Yuan Y., Wu X., Liu A., Wang J., Yang S., Liu B., Kong Y., Wang L., Zhang K. (2022). Excitatory SST Neurons in the Medial Paralemniscal Nucleus Control Repetitive Self-Grooming and Encode Reward. Neuron.

[101] Yeshurun S., Rogers J., Short A.K., Renoir T., Pang T.Y., Hannan A.J. (2017). Elevated Paternal Glucocorticoid Exposure Modifies Memory Retention in Female Offspring. Psychoneuroendocrinology.

[102] Wang L., Cai Y., Fan X. (2018). Metformin Administration during Early Postnatal Life Rescues Autistic-Like Behaviors in the *BTBR* T+ Itpr3tf/J Mouse Model of Autism. Front. Behav. Neurosci..

[103] Seibenhener M.L., Wooten M.C. (2015). Use of the Open Field Maze to Measure Locomotor and Anxiety-like Behavior in Mice. J. Vis. Exp..

[104] Eissa N., Jayaprakash P., Stark H., Łażewska D., Kieć-Kononowicz K., Sadek B. (2020). Simultaneous Blockade of Histamine H3 Receptors and Inhibition of Acetylcholine Esterase Alleviate Autistic-Like Behaviors in *BTBR T+ Tf/J* Mouse Model of Autism. Biomolecules.

[105] Prut L., Belzung C. (2003). The Open Field as a Paradigm to Measure the Effects of Drugs on Anxiety-like Behaviors: A Review. Eur. J. Pharmacol..

[106] Javed H., Azimullah S., Abul Khair S.B., Ojha S., Haque M.E. (2016). Neuroprotective Effect of Nerolidol against Neuroinflammation and Oxidative Stress Induced by Rotenone. BMC Neurosci..

[107] Eissa N., Awad M.A., Thomas S.D., Venkatachalam K., Jayaprakash P., Zhong S., Stark H., Sadek B. (2022). Simultaneous Antagonism at H3R/D2R/D3R Reduces Autism-like Self-Grooming and Aggressive Behaviors by Mitigating MAPK Activation in Mice. Int. J. Mol. Sci..

